# Sustained splenic contraction after daily cocaine administration in rats

**DOI:** 10.1371/journal.pone.0252853

**Published:** 2021-06-04

**Authors:** Moeka Nomura, Kana Unuma, Toshihiko Aki, Koichi Uemura

**Affiliations:** Department of Forensic Medicine, Graduate School of Medical and Dental Sciences, Tokyo Medical and Dental University, Tokyo, Japan; Indiana University Purdue University at Indianapolis, UNITED STATES

## Abstract

The purpose of this study is to examine the effect of repeated cocaine administration on the whole body of rats. Rats (male, 6 weeks old, Sprague Dawley) were injected intraperitoneally with cocaine (50 mg/kg) once a day for 1, 3 or 7 days, and major organs (heart, liver, lung, brain, kidney, spleen) were excised from the sacrificed animals. During autopsy, we found a reduction in spleen size, but not other organs, in cocaine-administered rats as compared to control rats. This reduction became to be noticed at 3 day and easily perceived at 7 day. No marked changes were observed in other organs examined. H&E and EMG staining showed a tendency for a decrease in the number of red blood cells (RBCs) as well as an increase in collagen fibers in the spleens of rats treated repeatedly with cocaine. Transcriptome analysis indicated that repeated cocaine administration depletes RBCs from the spleen. Immunoblot analysis showed that cocaine increases the phosphorylation of myosin light chain (MYL) as well as the levels of transgelin, both of which are involved in the contraction of myofibrils. Collectively, these results show that repeated cocaine administration results in sustained contraction of the spleen, which leads to the release of RBCs from the spleen into circulation.

## Introduction

Due to its euphoric effects, cocaine is distributed in the world as an illegal recreational drug [1]. Cocaine induces feelings of intense pleasure and reward in humans through the excitement of the reward center in the central nerve system (CNS) [2]. This is a consequence of the inhibitory effects of cocaine on dopamine reuptake from the synaptic cleft and the resultant increase in dopamine levels [2]. Cocaine also inhibits the reuptake of other catecholamines, such as adrenaline and noradrenaline, into the adrenergic nerve [3, 4]. Therefore, cocaine works not only as an activator of the CNS reward system, but also a vasoconstrictor in the vascular system [5, 6]. Indeed, most cocaine abusers who visit emergency departments mention cardiovascular complaints as the reason for visiting [5]. On the other hands, it has been widely recognized that all major organs can be affected by cocaine abuse [7]. In addition to above mentioned harmful effects of cocaine on CNS and cardiovascular system, for example, pulmonary complications such as edema could be produced by cocaine [8]. Furthermore, though rare in cocaine abuse-related cases as compared to cardiovascular complications, cocaine can cause abdominal complications [9], which may lead finally to splenic rupture [10–12], gastrointestinal perforation [13], ischemic colitis [14, 15], acute renal failure [16, 17], and hepatocellular necrosis [18]. Bibliographic survey led us, first of all, to macroscopic examination as well as calculation of relative weights of heart, liver, lung, brain, kidney, and spleen in rats administered cocaine.

The spleen is a secondary lymphoid organ involved in the regulation of immunity. Splenic parenchyma consists mainly of red pulp as well as white pulp, the latter of which includes the marginal zone that discriminates between the white and red pulp [19, 20]. The white pulp area is where immune responses against blood-borne pathogens occur [19, 20]. The red pulp, which is abundant in red blood cells (RBCs), serves as a filter to discriminate senesced RBCs from healthy RBCs; old as well as damaged RBCs are collected and destroyed in the red pulp, and the iron inside them is salvaged and stored for recycling [19, 20]. Since the spleen is a relatively large organ and extremely rich in RBC, it is considered to act as a reservoir of RBCs [21, 22]. During oxygen deficiency caused by diving [23], exercise [24], high altitude [25, 26], or sleep apnea [27], the spleen contracts to release RBCs into the central as well as peripheral circulation for oxygenation [22]. This RBC supply through splenic contraction is considered to help cardiac output [21].

For this study, we adopted repeated cocaine administration, which mimics the “binge” administration frequently observed in human abusers [28]. When we investigated multiple organ injury caused by repeated cocaine administration, we found a reduction in the volume of the spleen to be the most apparent finding in the gross pathological examination of major organs. Shrinkage of the spleen was found to be the result of sustained splenic contraction caused by repeated cocaine administration.

## Materials and methods

### Animals and cocaine treatments

All animal experiments were approved by the Institutional Animal Care and Use Committee of Tokyo Medical and Dental University. Rats (male, 6 weeks old, Sprague-Dawley, 〜220 g body weight, obtained from Charles River Laboratories Japan, Inc., Yokohama, Japan) were separated into 6 groups (n = 4 in each groups) and administered cocaine (cocaine groups) or saline (control groups). To optimize environmental conditions, rats were housed in a room with controlled temperature (25±1°C), relative humidity (60 ±5%), and a 12 h light/dark cycle. Bedding-change frequency was twice a week. Rats were given free access to water and a certified diet (Oriental Yeast Co. Ltd., Japan). Animal health surveillance was provided twice daily in the morning and evening. To assess animal health and well-being, we monitored the condition of rats over whole periods of time for negative affective states (e.g., loss of body weight), environmental conditions, bedding, feed, water, and housing. We dissolved cocaine hydrochloride (Shionogi & Co., Ltd., Osaka, Japan) in saline and administered it intraperitoneally (i.p.) to rats at a final dose of 50 mg/kg body weight once a day for 1–7 days. Control rats were injected with the same volume of saline. 24 hours after the last administration, the rats were sacrificed by an overdose of sodium pentobarbital (40 mg/kg, i.p.), and the hearts, right lungs, spleens, left kidneys, brains, and livers were removed immediately. The excised organs were weighed and stored at -20°C or -80°C for the later extraction of proteins or RNAs, respectively. Pieces of organs were fixed with 4% paraformaldehyde for tissue staining.

### Tissue staining

Spleen samples were embedded in paraffin, cut into slices at a thickness of 2.5 μm, affixed to glass slides, deparaffinized, and subjected to hematoxylin-eosin (H&E) and Elastica-Masson-Goldner (EMG) staining. The specimens were observed under a light microscope (AX-80, Olympus, Tokyo, Japan).

### Immunoblotting analysis

For western blot analysis, tissues were lysed in lysis buffer [320 mM sucrose, 10 mM Tris-HCl (pH 7.4), 1 mM EDTA-2Na, 10 mM NaF, 1 mM Na_3_VO_4_, and protease inhibitor cocktail (Complete, Roche, Mannheim, Germany)]. Equal amounts of spleen lysates were subjected to SDS-PAGE. Proteins separated in the gels were transferred to PVDF membranes, blocked with TBS-Tween [150 mM NaCl, 10 mM Tris-HCl (pH7.4), 0.05% Tween 20] containing 5% skim milk, and incubated with primary antibodies: anti-rat collagen type 1 (#AB755P, Merck Millipore, USA), anti-E cadherin (610181, BD Biosciences, USA), anti-cleaved caspase 3 (#9661, Cell Signaling technology, USA), anti-Thr18/Ser19-phosphorylated myosin light chain 9 (p-MYL, sc-12896, Santa Cruz Biotechnology, inc., USA), anti-myosin light chain (total MYL, sc-365243, Santa Cruz Biotechnology, inc., USA), anti-actin (A2066, Sigma-Aldrich), and anti-glyceraldehyde-3-phosphate dehydrogenase (#MAB374, Merck Millipore, USA). The samples were then incubated further with a peroxidase-bonded anti-IgG secondary antibody (Promega, USA), and the antigens were visualized using ECL reagents (Thermo Fisher Scientific, USA). Membrane images were obtained by LuminoGraph Ⅲ image capture (ATTO, Japan), and the luminescence levels were quantified by CS analyzer 4 image analyzing software (ATTO).

### Transcriptome analysis

For transcriptome analysis, total RNAs were extracted from rat spleen tissues using TRIzol reagent (Thermo Fisher Scientific). The total RNA was further purified using an RNeasy Mini Kit (Qiagen, USA), which includes removal of genomic DNA through digestion with DNase I. The purified total RNA was examined for integrity on a BioAnalyzer (Agilent Technologies), and hybridized to Clariom^TM^S array (Thermo Fisher Scientific). The results were deposited in the GEO database (https://www.ncbi.nlm.nih.gov/geo/, accession number GSE167238) and analyzed using the Microarray Data Analysis Tool (Filgen Inc, Nagoya, Japan) and the DAVID functional annotation tool (https://david.ncifcrf.gov/summary.jsp).

## Results

### Effects of repeated cocaine administration on internal organ weights and spleen volume in rats

**Fig 1:**
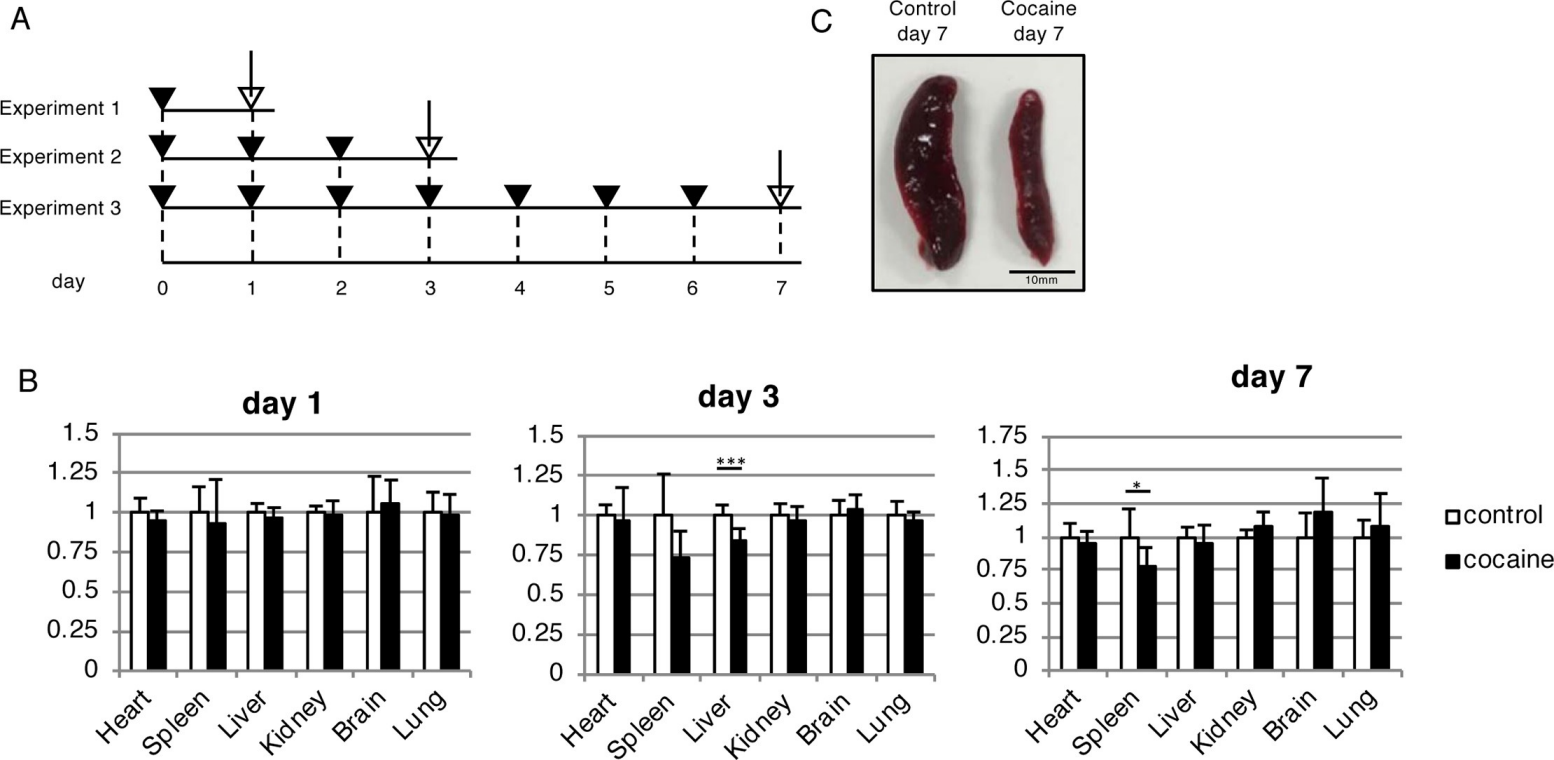
Changes in relative organ weights and reduction in spleen size in rats administered cocaine. (A) Timeline of experimental procedures. Rats were administered cocaine (50 mg/kg) once daily, and sacrificed on day 1, day 3, or day 7. Filled arrowheads indicate cocaine administration. Open arrows indicate autopsy and sample collection from sacrificed rats. (B) Relative weights of indicated organs per body weights. In each graph, the mean relative weight of the control group was set to 1. Graphs show the means and S.D. of 6 samples. *P < 0.05 versus control by Student’s *t*-test. (C) Reduction in splenic volume in rats repeatedly administered cocaine (day 7).

We first examined the effects of single and repeated intraperitoneal injection of a fixed dose of cocaine (50 mg/kg) on the weights of the internal organ. Since cocaine abuse by teenagers have become a social problem, we used rats at their late adolescent stage (6-week-old). Male rats were used throughout this study. Given the sex differences in the cocaine abuse [29], the results may be different when female rats are used. For the single injection experiment (experiment 1), rats were administered cocaine (day 0) and sacrificed on the following day (day 1) (Fig 1A). For the repeated injection experiments (experiments 2 and 3), rats were administered cocaine on day 0, and then once daily on the next two (experiment 2) or 6 days (experiment 3) (Fig 1A). In all experiments, the rats were sacrificed one day after the final cocaine administration. We measured the weights of the heart, spleen, liver, left kidney, brain and right lung, and divided these by the body weight. Although we initially planned to excise pancreas too, we gave priority to remove spleen as its intact form; removal of pancreas sometimes requires sacrifice of spleen. In experiment 1, no obvious changes were observed in the relative weights of any organ in the cocaine group as compared to the control group (Fig 1B). Although there were several upwards or downwards tendencies in the relative weights of the liver, kidney, brain, and lung (for example, significant decrease of liver weight observed at day 3), these trends were relatively small and kept on fluctuating over time (Fig 1B). In contrast we observed a tendency toward a decreased relative weight of spleen, which was persisted from day 3 to day 7 (Fig 1B). We also found an apparent reduction in spleen volume as the most obvious gross anatomical change in the cocaine group (Fig 1C). Therefore, we focused on changes in the spleen following repeated cocaine administration throughout this study. It should be noted that our current results did not necessarily indicate that cocaine did not affect relative weights of kidney, brain, heart, and lung in our experimental setting. Further study may reveal significant changes in these organs.

### Histological alterations in spleen by repeated cocaine administration

**Fig 2:**
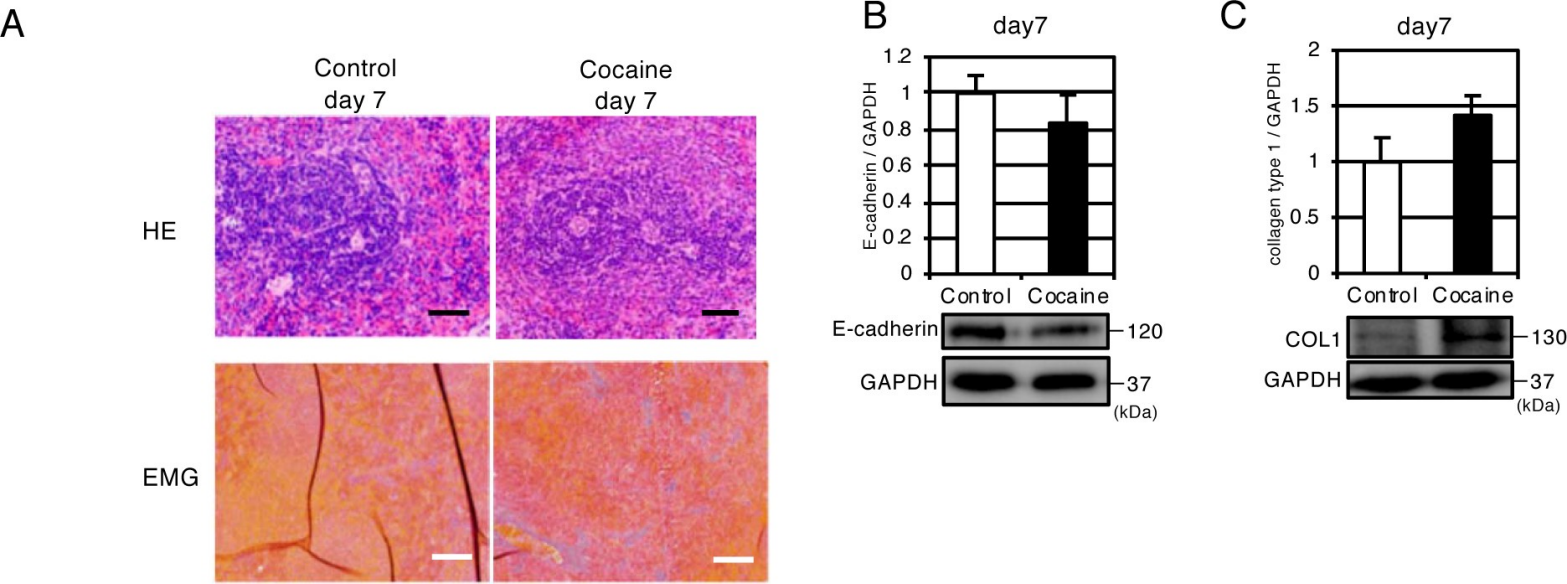
Histochemical and immunoblot analysis of spleen from rats repeatedly administered cocaine (day 7). (A) Hematoxylin and eosin (H&E) and Elastica Masson-Goldner (EMG) staining of spleen. Black and white bars indicate 50 μm and 100 μm, respectively. (B-C) Immunoblot analysis of E-cadherin (B) and collagen type 1 (C). GAPDH levels served as the internal control. Graphs show the means and S.D. of 6 samples. *P < 0.05 versus control by Student’s t-test.

To examine the histological differences between the spleens of control rats and those in the cocaine group (day 7), tissue samples were stained with hematoxylin-eosin (H&E) and Elastica-Masson-Goldner (EMG). In H&E stained sections, the cell density in the white pulp including the marginal zone seemed to be reduced in the cocaine group as compared to the control group (Fig 2A). Western blot analysis showed that levels of E-cadherin, which is involved in cell-cell adhesion [30], was decreased in the cocaine group as compared to the control group (Fig 2B). In the EMG stained samples, the number of collagen fibers (stained green) seemed to increased, while the relative abundance of RBCs (stained orange) decreased in the cocaine group as compared to control group (Fig 2A). The increase in collagen fibers was confirmed by an increase in the level of collagen type 1, which was observed by western blot analysis (Fig 2C). Taken together, these results confirmed that cocaine administration for 7 days induces not only atrophy, but also other pathological changes, such as the loosening of cell-cell adhesion and a subsequent increase in the number of collagen fibers in the spleen.

### Transcriptional profiling of splenic changes caused by repeated administration of cocaine

**Fig 3:**
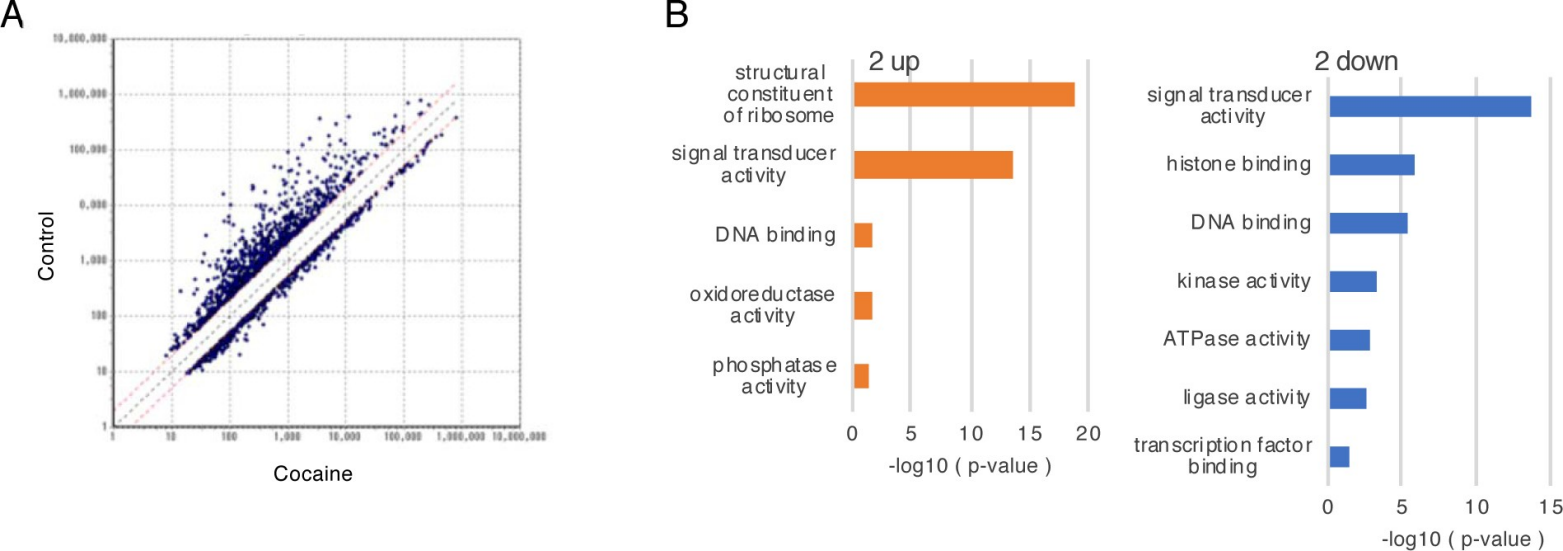
Transcriptional profile of spleen from rats repeatedly administered cocaine (day 7). Total RNAs from spleen (day 7) were subjected to DNA microarray and subsequent gene ontology (GO) analysis. (A) A scatter plot analysis of transcripts that showed greater than 2-fold responses to cocaine. The data below the lower dotted line indicate a greater than 2-fold increase caused by cocaine. The data above the upper dotted line indicate a greater than 2-fold decrease caused by cocaine. (B) Molecular function categories (Gene Ontology terms, GO) identified by Microarray Data Analysis Tool as being affected by cocaine in the spleen. P-values were calculated by comparison to the control group.

To obtain insight into the observed changes in spleen caused by repeated cocaine administration (Figs 1 and 2), we performed transcriptome analysis. Total RNAs from the control and cocaine groups (day 7) were subjected to DNA microarray analysis that resulted in the identification of 464 and 1047 transcripts that were increased and decreased more than 2-fold, respectively, in the cocaine group as compared to the control group (Fig 3A). Biological process and pathway analysis based on the GO terms of the Microarray Data Analysis Tool showed “structural constituent of ribosome” and “signal transducer activity” as the processes most significantly influenced by cocaine among the upregulated and downregulated genes, respectively (Fig 3A). To obtain further information, KEGG pathways significantly affected by repeated cocaine administration were identified using DAVID software. We found that among the top 20 processes whose transcripts were significantly downregulated by cocaine, 5 processes were restricted to RBC functions (Table 1). Moreover, among the top 50 genes whose expressions were decreased by cocaine, 22 genes were related to the function of RBCs (Table 2). Considering the fact that spleen contains abundant RBCs, these results suggest that repeated cocaine administration for 7 days results in the ejection of RBCs from the spleen, which might be related to the reduction in splenic volume.

**Table 1:** Categories related to RBC function that are significantly downregulated by cocaine.

GO.ID	Term	Count ^a^	P-Value
GO:0006783	heme biosynthetic process	14	2.93E-16
GO:0006779	porphyrin-containing compound biosynthetic process	9	1.48E-10
GO:0030218	erythrocyte differentiation	14	1.76E-07
GO:0048821	erythrocyte development	9	6.57E-06
GO:0010039	response to iron ion	9	1.29E-05

^a^ The number of genes downregulated by cocaine and classified into the category.

**Table 2:** List of 22 genes related to RBC that are included among the top 50 genes downregulated by cocaine.

EntrezGene ID	Gene Symbol	Gene Description	Ratio (cocaine/control)
362202	Epb42	erythrocyte membrane protein band 4.2	0.004894405
65207	Rhag	Rh-associated glycoprotein	0.010322416
314251	Sptb	spectrin, beta, erythrocytic	0.011312488
289257	Spta1	spectrin, alpha, erythrocytic 1	0.012354514
24779	Slc4a1	solute carrier family 4 (anion exchanger), member 1	0.015326618
298485	Ermap	erythroblast membrane-associated protein	0.016033279
25475	Lgals5	lectin, galactose binding, soluble 5	0.021909885
---	Gypa	glycophorin A	0.023699113
60414	Rhd	Rh blood group, D antigen	0.027341142
306570	Ank1	ankyrin 1, erythrocytic	0.029378987
361338	Fech	ferrochelatase	0.030773674
294210	Trim10	tripartite motif-containing 10	0.037144138
25709	Hmbs	hydroxymethylbilane synthase	0.037605805
25240	Aqp1	aquaporin 1	0.037921865
25748	Alas2	5-aminolevulinate synthase 2	0.044641618
361439	Abcb10	ATP-binding cassette, subfamily B (MDR/TAP), member 10	0.049336537
361069	Dmtn	dematin actin binding protein	0.049893672
304666	Klf1	Kruppel-like factor 1 (erythroid)	0.051713904
29421	Urod	uroporphyrinogen decarboxylase	0.055682715
114511	Emb	embigin	0.056816553
304775	Dyrk3	dual-specificity tyrosine-(Y)-phosphorylation regulated kinase 3	0.057535695
64678	Tfrc	transferrin receptor	0.058454373

### Sustained contraction of spleen in repeated cocaine administered rats

**Fig 4:**
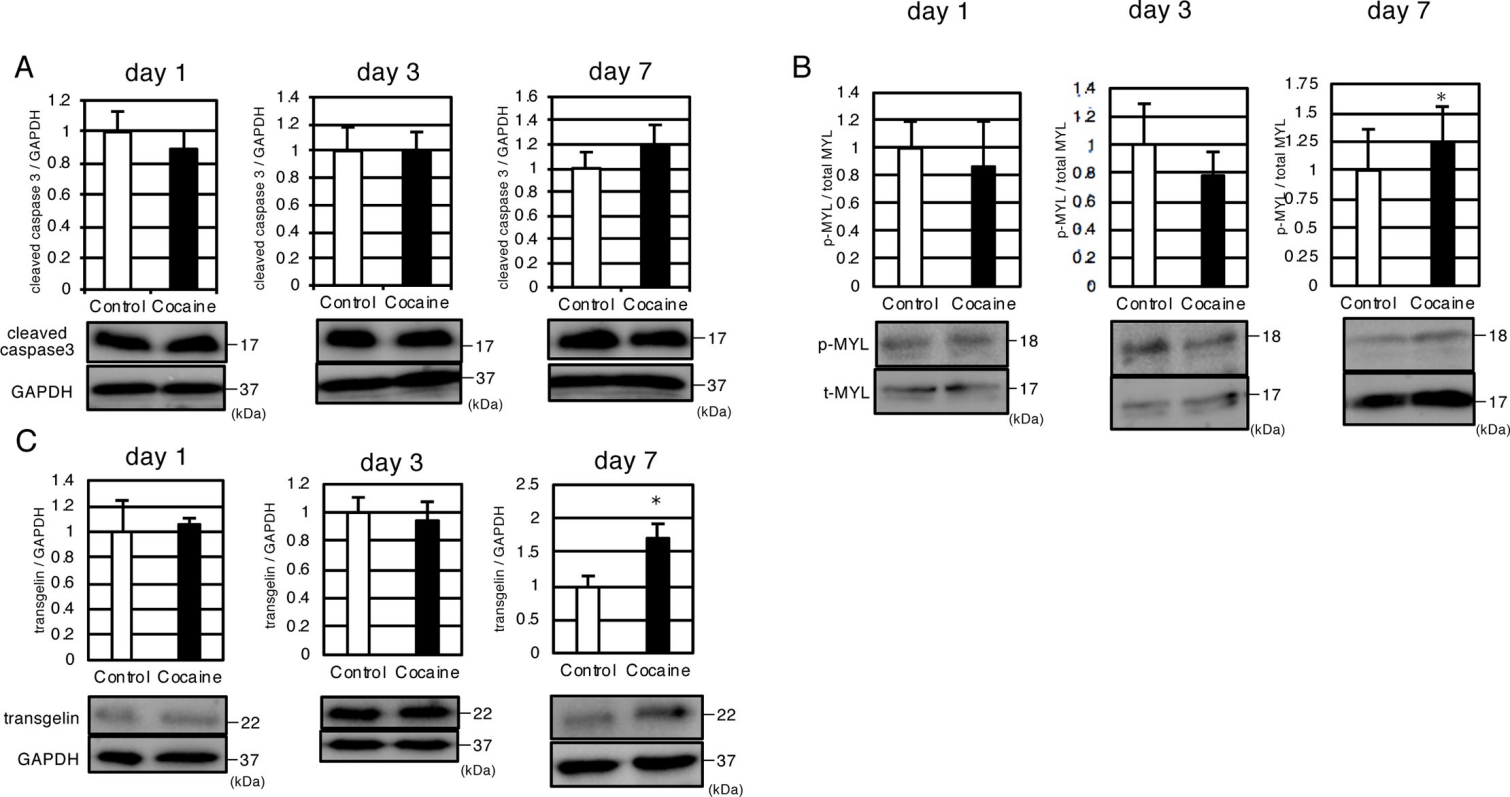
Repeated administration of cocaine upregulates contractile marker proteins, but not a marker of apoptosis in rat spleen. Immunoblot analysis of cleaved caspase 3 (A), phosphorylated as well as total MYL (B) and transgelin (C) in the spleens of rats administered cocaine. GAPDH or actin levels served as internal controls. Graphs show the means and S.D. of 6 samples, except for phosphorylated MYL of the cocaine group of day 7 (5 samples). *P < 0.05 versus control by Student’s *t*-test.

We next investigated whether the reduction in spleen size is a result of apoptosis or contraction. We first performed immunoblot analysis using an antibody against cleaved caspase 3, which is a marker of apoptosis [31]. No increase in the cleaved caspase 3 levels was observed in any of the cocaine groups (1-, 3-, or 7-day experiments; Fig 4A). We also checked for markers of other kinds of cell death such as necroptosis (RIP1) [32], pyroptosis (GSDMD) [33] and ferroptosis (GPX4) [34] by immunoblot analysis, and detected no significant changes in any of these proteins (S3 Fig). Next we analyzed for contractile marker proteins: myosin light chain, a cytoskeleton protein that regulates contraction in smooth muscle and non-muscle cells and is activated by phosphorylation [35], and transgelin, which contributes to smooth muscle contraction in a calcium ion-independent manner [36]. In contrast to cleaved caspase 3, we detected a tendency towards an increase (day 3) and a significant increase (day 7) in the phosphorylation of the myosin light chain (MYL) in the cocaine groups (Fig 4B), suggesting increased contraction of the spleen by repeated cocaine administration. Splenic contraction was further evidenced by an upregulation of transgelin in the cocaine group (day 7, Fig 4C). Taken together, these observations indicate that the administration of cocaine for 7 days results in sustained contraction of the spleen.

## Discussion

In this study, we have shown that repeated cocaine administration leads to sustained contraction of spleen in rats. Roughly speaking, there were tendencies that weights of other organs such as liver, kidney, brain, and lung decreased after 3 days cocaine administration and increased after 7 days. However, these changes were relatively small compared to that of spleen and there were not certain tendencies in the observed changes of organ weights except spleen.

A reduction in spleen size might occur through 1) autosplenectomy, which might include apoptosis and other forms of cell death [37], and 2) splenic constriction [22]. Although the possible involvement of the former has not been eliminated completely in this study, our current results showing the upregulation of the phosphorylated active form of myosin (Fig 4B) and the decrease in RBC in the cocaine administered spleen (Tables 1 and 2) strongly suggest that atrophy is a result of sustained splenic contraction. It should be noted that splenic contraction and autosplenectomy might be related to each other: excessive contraction may lead to vascular constriction, which can result in infarction as well as ischemia. Further studies are needed to evaluate the relationship between splenic constriction and autosplenectomy.

An MRI-based human study has shown that a single administration of cocaine (0.4 mg/kg) results in transient splenic contraction, during which the reduction in splenic volume reached approx. 20% around 10 min after administration, and recovered to the basal level within 35 min [38]. Since we excised organs including spleen one day after the last administration of cocaine, splenic constriction appears to last for at least one day after daily cocaine administration for 7 days. Therefore, our findings indicate that transient splenic contraction by cocaine may become chronic after repeated administration for 7 days. Since the spleen is considered to serve as a reservoir of RBC for oxygenation of the central as well as peripheral tissues during periods of high oxygen demand as described in “Introduction”, these results suggest the possible involvement of the organ in oxygenation against the challenge of cocaine.

In addition to sustained contraction, we also observed a tendency towards fibrosis in the spleens of cocaine-administered rats; EMG staining indicated a slight increase in collagen fibers, and immunoblot analysis also showed an upregulation of collagen type 1 in the cocaine groups as compared to the control group (Fig 2C). We currently do not have an idea concerning the exact cause and consequences of splenic fibrosis. Splenic vascular constriction may lead to infarction as well as ischemia, which might be a cause for the tendency towards fibrosis.

Throughout this study, we used male rats aged 6-week-old as of the time starting experiments. Six-week-old rats are in their late adolescent stage, in other words in their pre-adult stage [39]. It has been shown that sex and age should have substantial effects on the outcomes of cocaine addiction both in humans and animal models [29, 40]. For example, stronger adverse effects of cocaine on cognitive function are observed in adolescent-onset cocaine exposure than in adult-onset exposure [40]. Concerning to the sex differences for the sensitivity to cocaine addiction, estradiol is supposed as a key factor of this difference [41]. Since estrogen is also implicated in the regulation of vascular function [42], there may be a difference in cocaine-induced splenic contraction between male and female rats.

In conclusion, we have demonstrated that after daily administration of 50 mg/kg cocaine for 7 days, splenic constriction becomes sustained for at least 1 day. Sustained splenic constriction might be a reasonable result of repeated transient contraction. The pathophysiological implications of this phenomenon might shed light not only on the splenic dysfunction, but also the multiorgan failure caused by the abuse of cocaine.

## Supporting information

S1 Fig The full length blots of Fig 2. (PDF)

S2 Fig The full length blots of Fig 4. (PDF)

S3 Fig Repeated administration of cocaine does not affect pyroptosis, necroptosis, and ferroptosis markers in rat spleen. Immunoblot analysis of phosphorylated-RIP1 (pRIP1), GSDMD, and GPX4 in the spleens of rats administered cocaine. p30 fragment of GSDMD was not observed both in control and cocaine groups. Actin levels were served as internal control. Graphs show the means and S.D. of 6 samples.
(PDF)
