# Supplementary figures and images for: Sustained splenic contraction after daily cocaine administration in rats
Source: PLoS One. 2021 Jun 4;16(6):e0252853. doi: 10.1371/journal.pone.0252853 (PMC8177630; doi:10.1371/journal.pone.0252853)

# S1 Fig

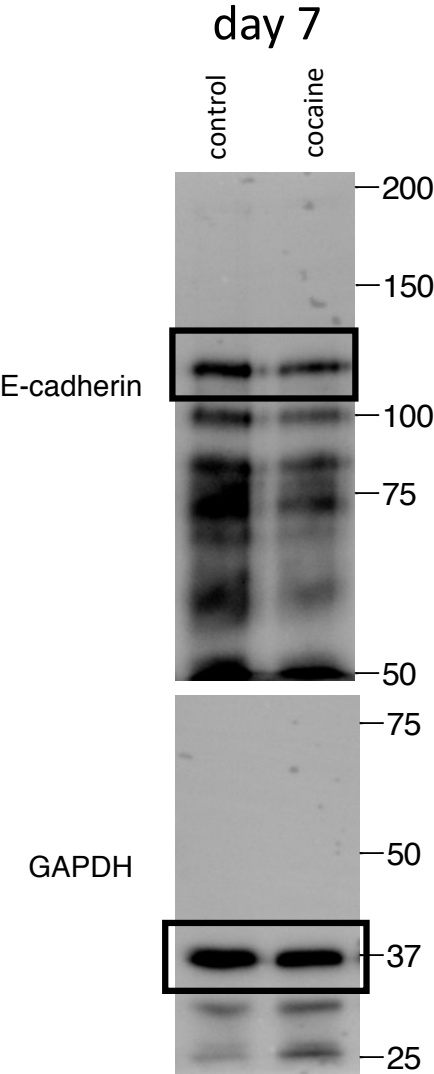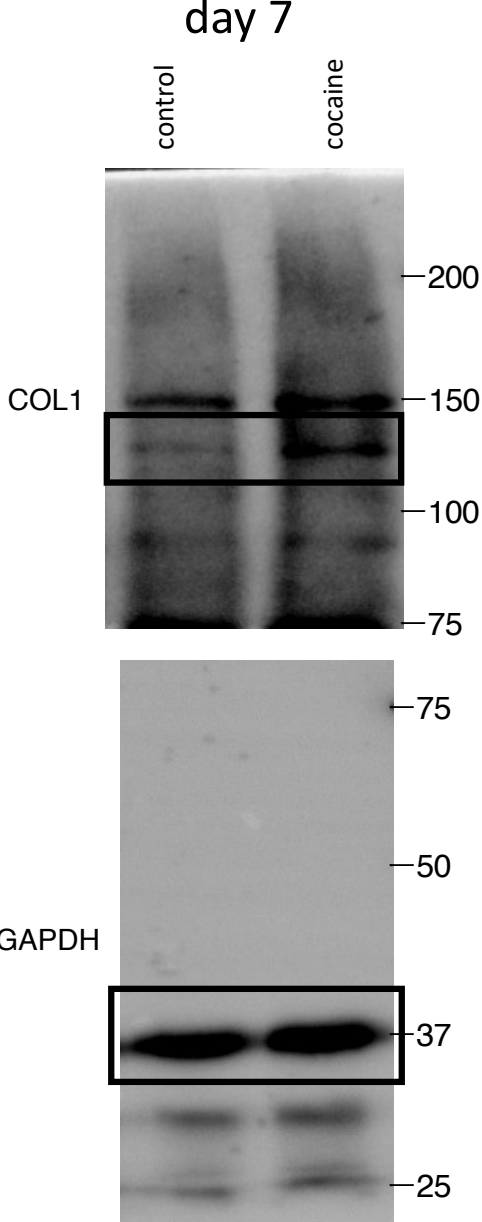

Supplement: S1 Fig — (PDF) [file pone.0252853.s001.pdf]

# S2 Fig

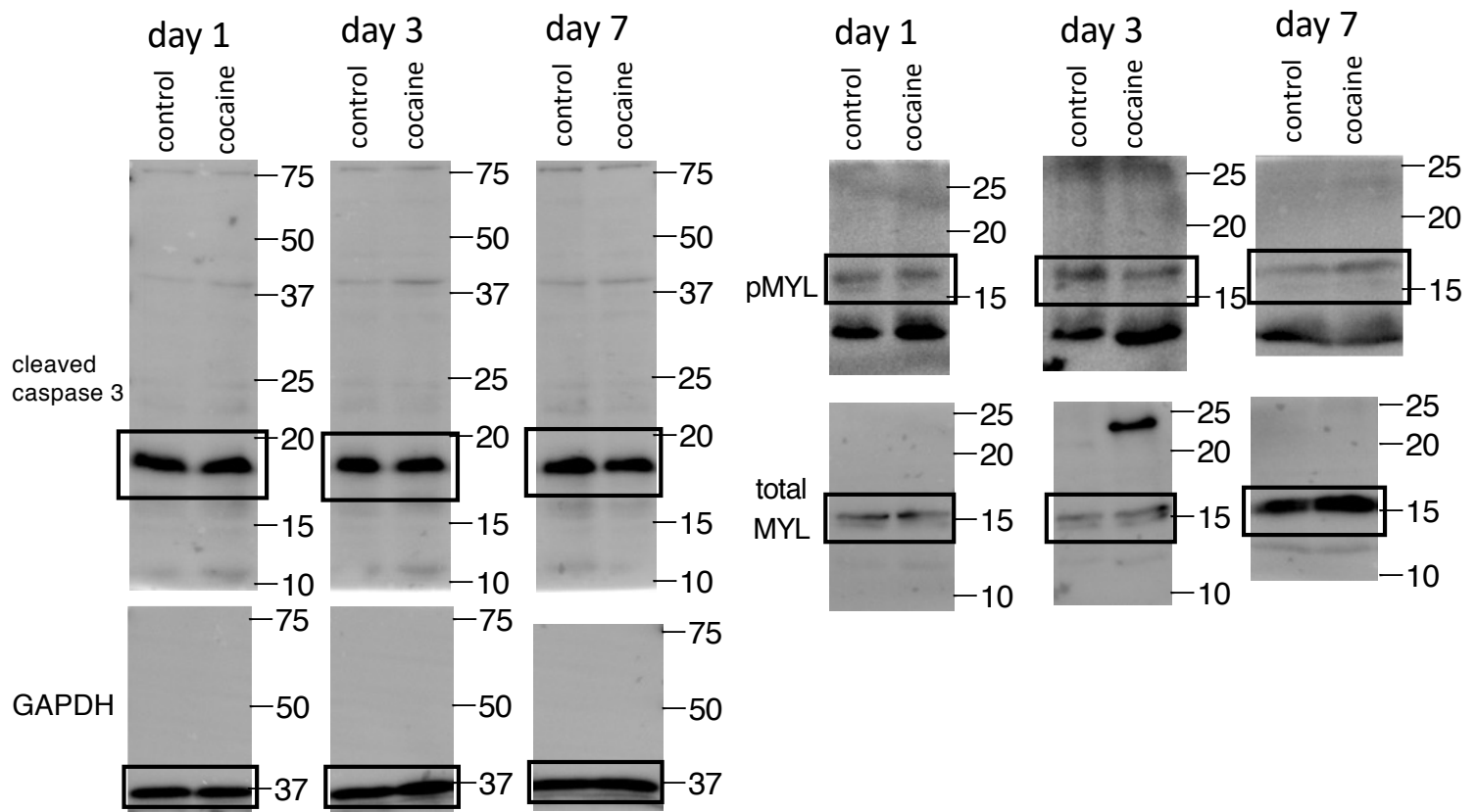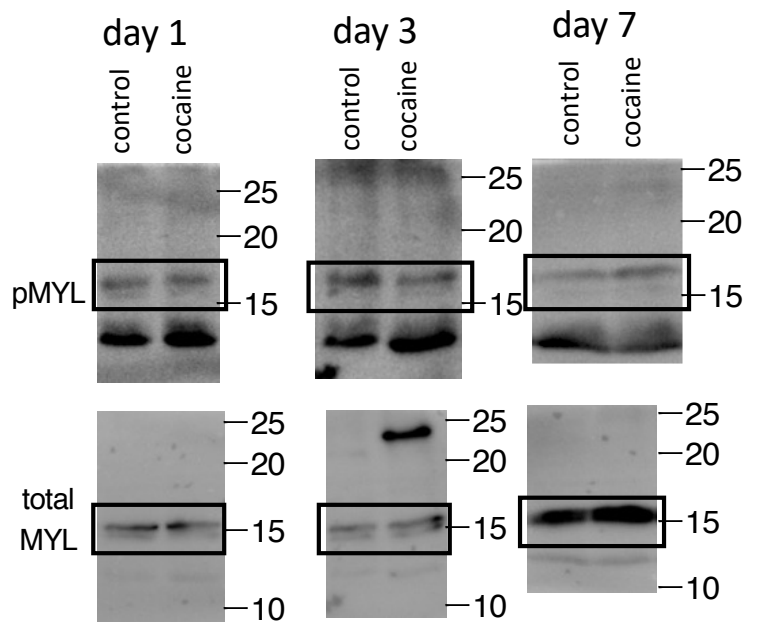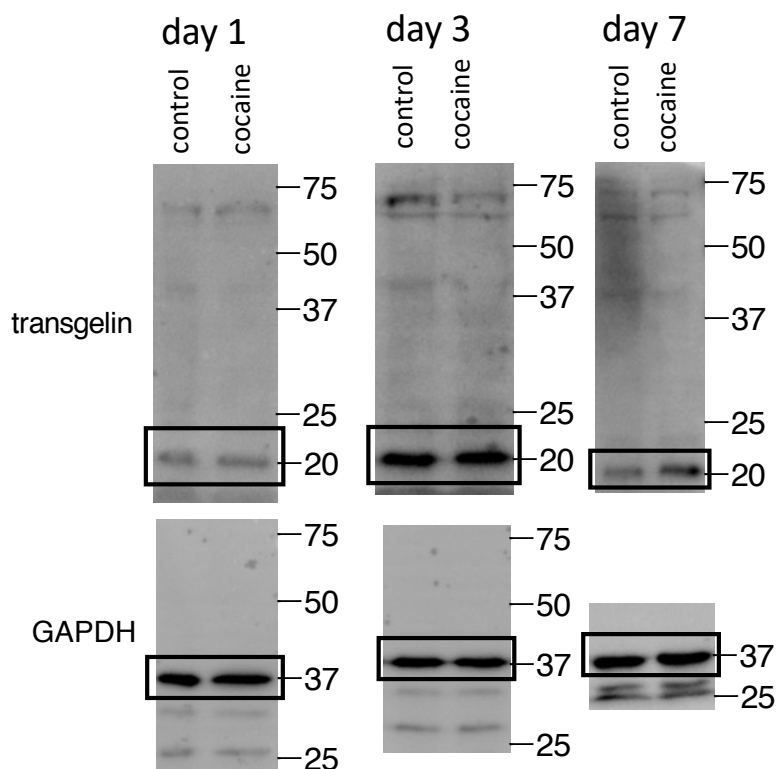

Supplement: S2 Fig — (PDF) [file pone.0252853.s002.pdf]

# S3 Fig

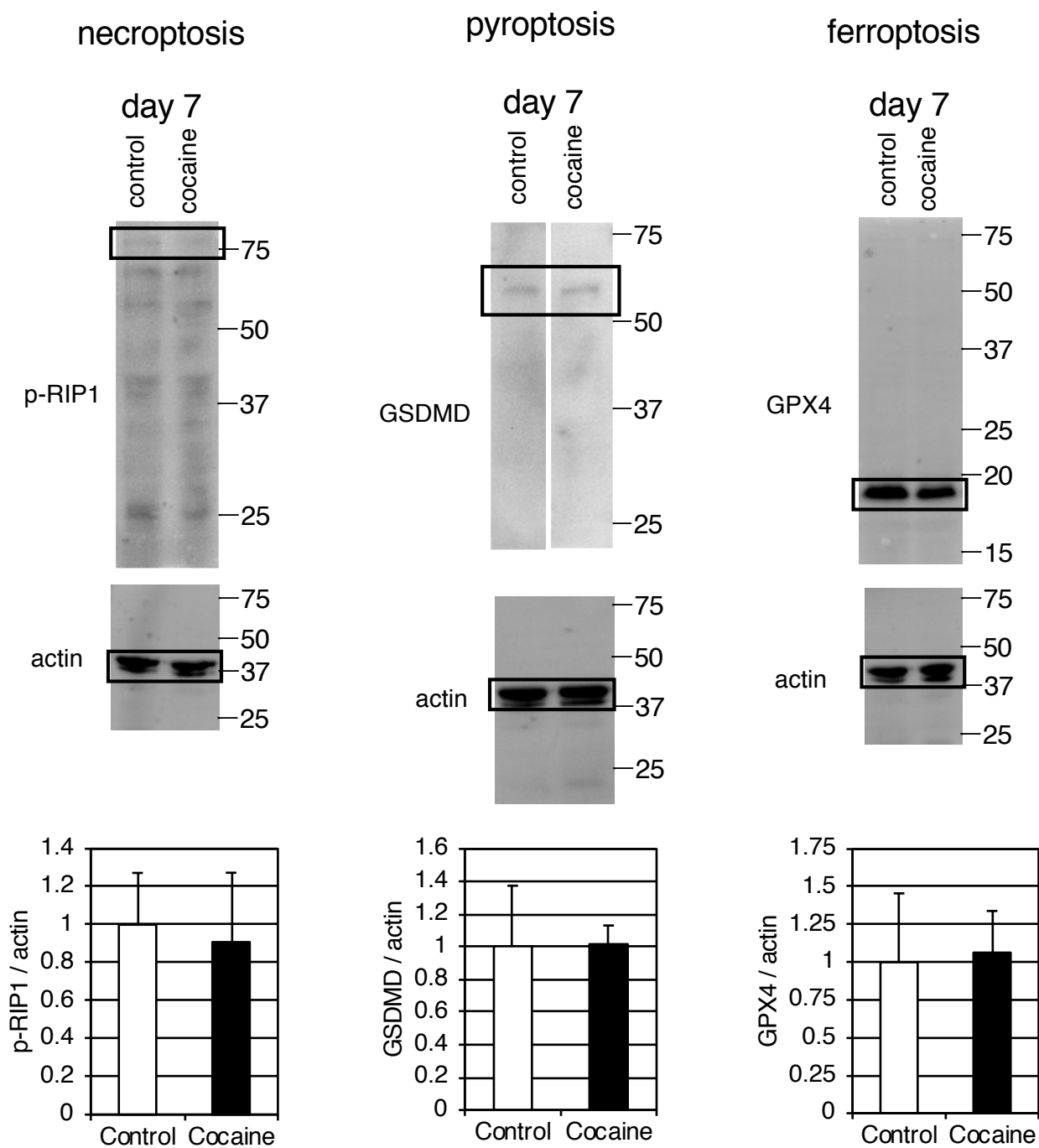

Supplement: S3 Fig — Immunoblot analysis of phosphorylated-RIP1 (pRIP1), GSDMD, and GPX4 in the spleens of rats administered cocaine. p30 fragment of GSDMD was not observed both in control and cocaine groups. Actin levels were served as internal control. Graphs show the means and S.D. of 6 samples. (PDF) [file pone.0252853.s003.pdf]
